# Migration and Regional Differences in Access to Local Family Networks Among 60-year olds in Sweden

**DOI:** 10.1007/s12062-015-9117-z

**Published:** 2015-03-11

**Authors:** Emma Lundholm

**Affiliations:** 1 Centre for Population Studies, Umeå University, Umeå, Sweden; 2 Department of Geography and Economic History, Umeå University, Umeå, Sweden

**Keywords:** Local family networks, Internal migration, Intergenerational families, Regional differences in ageing, Sweden

## Abstract

Regional variations in access to local family networks has implications for future care burdens in different regions as well as the living conditions for both older and younger generations. The geographical distance between family members is a long-term consequence of accumulated migration and non-migration undertaken by the individual as well as other family members. This study contributes to this subject through offering a description of regional disparities in the access to local family networks among 60-year olds in Sweden. Additionally, this paper aims to analyse this pattern as an outcome of long-distance migration processes. The empirical study is based on Swedish register data, with a focus on 60-year olds in Sweden, linking them to their adult children, siblings and parents as well as in-laws. The dataset includes total population, where it is possible to identify family networks in their geographical context on various geographic scales, down to a neighbourhood level. As expected, results indicate that families in metropolitan areas are the most concentrated geographically while the left behind parent, embedded in a local network in their own and older generation, is a small category in urban areas but quite common in some rural municipalities. It is also shown that access to local family networks not only varies on a broad rural–urban scale but also locally, between neighbourhoods within metropolitan areas.

## Introduction

In an ageing society there are substantial regional variations in terms of the number of elderly people in relation to people of working age. This has led to a concern about potential future support rates on local and regional level. Alongside the uneven geographical distribution of the elderly versus younger individuals, there are also regional variations to the access of local family networks among the elderly when comparing urban and rural areas. The need for formal care is potentially higher if there is less informal care, and in regions where few elderly have a local family network, pressure increases on the public sector to provide support. Sweden is known to be a strong welfare state, but the ageing population is putting a strain on public sector provision of care. It has been claimed that elderly care is in a process of re-familisation where families need to step in when the tax-paid elderly care is declining (Sundström et al. 2006; Szebehely and Trydegård 2012). The issue of local family networks is not only relevant in relation to elderly care, but are a potential resource for both older and younger generations (Hjälm 2011; Mulder and van der Meer 2009). This study focuses on the regional differences of proximity between family members and the demographic processes that produce geographic variation in access to local family networks among the elderly. It pays special attention to how this pattern is shaped by migration and non-migration. Thereby this study adds to the literature on how family geographies evolve as an outcome of mobility (Duncan and Smith 2002; Smith 2011). In this case, the family landscape described includes family members outside of the household, across generations.

The geographical distance between family members is the result of accumulated migration and non-migration for all generations throughout the lifespan, a consequence of family members staying close, moving away or moving closer to one another. Migration is highly selective. Young people are more mobile than older people and the highly educated are also more migratory. The migration pattern on an aggregated level makes an imprint on family structures on the regional level. For instance, the typical pattern for urbanization involves older generations staying in rural areas while the younger generations start their families in an urban area. For the next generation, those born in the urban areas are more likely to stay within close proximity to their parents. Changes in population settlement patterns influence what we can expect from the future density of local family networks, and hence access to family support networks.

The empirical study in this paper is based on Swedish register data, covering the total population, where it is possible to identify family networks in their geographical context on various geographic scales, down to neighbourhood level. In the data it is possible to identify the residential location of parents, children and siblings, including in-laws in terms of partner’s parents and siblings.

The aim of this study is to describe regional disparities in the access to local family networks, through studying a snapshot of the extended family networks of 60-year olds in Sweden. . Additionally, this paper aims to analyse this pattern as an outcome of long-distance migration processes. This paper poses the following research questions: What access do people have to local family networks in rural and urban regions in Sweden? How are these patterns related to migration processes on individual and aggregated levels?

## Background: Family Networks and Migration

The informal support that families provide is important for the well-being of both the older and younger generations (Bengtson 2001). The local family network should be seen as a resource for all ages in which the elderly members make a significant contribution. Studies in Sweden and in Europe have shown that the older generation provides more financial and functional support to the younger generation, rather than the other way around (Albertini et al. 2007; Halleröd 2006; Hoff 2007). Although Sweden is known to have a strong welfare state, informal support is an important addition to the formal support provided by the welfare state in terms of the care provided to the elderly, the sick, and towards the care of children (Sundström, et al. 2006; Szebehely 2006). Geographical proximity is an important factor for the frequency of family contact and support (Rainer and Siedler 2012; Scharf 2001).

Intergenerational exchanges are not necessarily extensive in one cross section of life, but rather in a life course perspective. Parents and children are sensitive to each other’s needs and will help when those needs arise, such as during important instances like divorce, widowhood, and sickness (Chan and Ermisch 2011). Help is often temporary but could be very important during a specific passage in life. Chan and Ermish emphasise that the latent family support should not be neglected and that geographic proximity is crucial.

The family network changes over the life-course, individuals are not only affected by their own life-course events but also events in the family. It is therefore valuable to adopt what Elder calls a’family life-course perspective where the linked lives of family members are taken into consideration’ (Elder 1994). New family members are born while other family members die. Households are formed and in-laws become new additions to families, and in some cases households and extended families are dissolved by separation and divorce. In addition to these transformations in the family network, the geography of the network can be modified by migration when family members move closer or further apart. Despite all these potential adjustments, which can be fundamental and dramatic events for the individual, it is important to acknowledge the stability of family networks. The focus for this paper is an age group in a phase of life with relatively modest changes in their family networks, especially when considering geographical proximity (Svensson et al., Family life course and the timing of women’s retirement - a sequence analysis approach. Population, Space and Place, (forthcoming)).

Several studies have explored the geographical distances between the elderly and their adult children, (Fors and Lennartsson 2008; Hank 2007; Malmberg and Pettersson 2008; Michielin and Mulder 2007; van der Pers and Mulder 2013). There is also now growing literature on the role of siblings (Blaauboer et al. 2013; van Gaalen et al. 2008; Voorpostel et al. 2007, 2012). For most people, sibling relationships are the longest held relationships in life. Although siblings often don’t have daily contact, they are life long, permanent members of each other’s family networks and can potentially step in as an important source for support and companionship in various phases of the life course. Giving and receiving support from siblings can reduce loneliness in middle and old age (de Jong Gierveld and Dykstra 2013). However, there are also findings that suggest that people who have experienced more severe life events such as abuse, mental problems or addiction have less contact and exchanges with their siblings (Voorpostel, et al. 2012).

Besides studies on the divergent demographic structures in rural and urban regions in an ageing population (eg. Walford and Kurek 2008), there are also studies on how the experience of growing old varies between living conditions in different geographic contexts. These often have a focus on health and health care provision for the elderly in rural and urban contexts (eg. Keating et al. 2011; Wanless et al. 2010). With regards to the elderly and their families in rural areas, there are (at least) two contradictory views. On the one hand is what Wenger (2001) calls a myth about a strong traditional family bond in rural areas, where the elderly are expected to have a more intimate relationship to their family members in rural areas compared to more individualistic life in urban areas. On the other hand there is a discussion about the elderly “left behinds” in rural areas, whose local family network is weakened by the migration of the younger generation. Studies of elderly left behinds are often situated in the developing world, where children have migrated as part of a national urbanization process or an international migration process (He and Ye 2013; Knodel and Saengtienchai 2007; Vullnetari and King 2008). The empirical evidence in western societies is ambivalent. Wenger did not (2001) find evidence that networks are stronger in rural areas in the UK, and according to Scharf (2001), intergenerational relations are characterised by frequent contact in both rural and urban regions in Germany.

Migration patterns are reflected in the configuration of family networks and shape structural and regional differences in the family landscape. For example, the typical urbanization process is when the younger generation moves into cities while the older generation remains in the countryside. This results in weakened family networks. This study, however, covers different spatial contexts since family networks are not only important in rural areas s Mulder and van der Meer (2009) point out, family support is important for people in both urban and rural areas. Therefore there is interest in investigating the urban context and the extent to which people that were born in cities live close to their family. Previous studies have shown that there are indeed differences between urban and rural areas. Van der Pers and Mulder (2013) found that parents living in urban areas are more likely to live close to their adult children compared to parents in more rural areas in the Netherlands. Similar results have has been found in Sweden by Malmberg and Pettersson (2008) suggesting that it is more common for elderly people living in rural areas to live far from their children while elderly city dwellers are more likely to have children living in the same region. However, when parents and children live in the same region, those in rural areas will live closer to each other when compared to urbanites (ibid.). Malmberg and Pettersson also shows how urbanization has made a long-term imprint on the intergenerational distances between cohorts, where urbanization generation ends up far away from their parents while younger generations are living with a shorter distance to their parents. Thus, this study further develops Malmberg and Petterson’s study by including a larger family network that includes siblings and the partner’s family. The current study could thereby contribute to an increased knowledgebase of the geographical contexts of family networks and the characteristics of regions with dense and less dense family networks.

Migration research recognises that family members can serve as attractions for migration. Compared to other European countries, the welfare system in the Nordic countries makes individuals less dependent on family for care and support. There are studies that suggest that location decisions and care giving choices are less associated with family structure in the Nordic countries as compared to other countries in Europe (Rainer and Siedler 2012). There are, however surveys indicating that moving closer to family and friends is a frequent motive for internal migration in Sweden (Lundholm et al. 2004). Adult children tend to move closer to their parents when they themselves have children (Pettersson and Malmberg 2009). This can be seen as an example of family networks that have an important function for young families and serve as a resource. Geographic proximity of family networks evolves, however, not through migration but through non-migration (Hjälm 2011). It can be an active choice to live near your family because you highly value the proximity to your family or you are dependent on the support provided by family. Staying close to family can also be something you feel compelled to do because of loyalty or expectations from family members. Staying close may also be less of an active choice and more a result of circumstances. Adult children may have never had reason to move, for example, after school they got a job right in the local community. Moving always involves a cost and a risk, so it may be more rational to stay. One factor that prevents migration is “insider advantages” which develop in one’s hometown through local knowledge and social networks (Fischer and Malmberg 2001).

Although the shaping of the family landscape is mainly the result of migration at a young age, such as the relocation that the current 60-year-olds did thirty or forty years ago, it also includes the migration that their children did at young ages, and the proximity of family members could also be a product of mobility at a later stages. However, the elderly are less likely to move home compared to younger people, and also for moving partly because of other reasons. A key motive for migration in this age group is to live closer to kin (Bell and Rutherford 2013; Pettersson and Malmberg 2009). Other motives are amenity motives. Retirees are an example of a group that have a higher propensity to choose rural areas as compared to others (Friedrich and Warnes 2000; Stockdale 2006). Migration which involves moving back to one’s birthplace is also a recognised type of migration for this age group (Lundholm 2012a, b).

## Study Design

The data used in the empirical study are Swedish register data from Statistics Sweden available through the Linnaeus database located at the Centre for Population Studies at Umeå University. It includes all 60-year-olds residing in Sweden in the years 2005–2009 (cohorts born 1945–1949). Total number is 633,166 individuals. By using the multigenerational register it is possible to link the individuals to adult children, partners, parents and siblings if they are residing in Sweden during the year of study. The linkage is very reliable for all persons born after 1932 in Sweden (95–100 %), including both biological and adopted children. Linkage of foreign born persons depends on age at immigration and if the families arrived together. Parents and children are linked if children were under age 19 at the time of immigration (Statistics Sweden 2010). Via this link the localization of the relatives can be used to calculate the distance between the 60-year olds and their relatives. In the database, localization is recorded on a 1 km*1 km grid. Regarding distance, the threshold of 50 km Euclidian distance was chosen as a cut-off as it can be regarded as a distance that allows for daily interaction.

The age of 60 has been chosen because by that age most of the children are in their late twenties and thirties and have left their most mobile period. Most long distance moves are undertaken at a younger age. After 30, migration propensity decreases significantly (Lundholm 2007). At the same time, it is still common for 60 year-olds to have a living parent and siblings. In order to capture the intergenerational characteristics of local networks, two indicators of presence for parents have been used. First, the location of any living parent (or parent in-law) of the sixty-year old., secondly the location of the parent the year of their passing going back 7 years in order to see if the present location of the 60 year old is the location where the parent lived at the time of their passing.

This study is designed to capture family network proximity over three generations: i) Own generation including siblings and partner’s siblings, ii) Older generation, including parents and partners parents and, iii) Younger generation including adult children. The study of density of family networks in these three dimensions could be related to general patterns of urbanization and counter-urbanization. In line with the purpose of the study: to explore how family geographies evolve as an outcome of mobility with special attention to how this pattern is shaped by migration and non-migration, the following classification is developed and used:

- [*Dense*] Dense local network in older and younger generations, children and parents and/or siblings
- [*Left behinds*] Dense local network in own and/or older generation but children far away
- [*Settlers*] No local family network in own and/or parents’ generation but close to children
- [*Solitary*] No local family network (children far away)
- [*Childless*/*Solitary*] No children, no local family network
- [*childless*/*dense*] No children, local family network in own and/or older generation

The distribution of 60-year olds in these categories was aggregated to municipalities (*N* = 290). The municipalities are divided into ten categories according to the definition by the Swedish Association of Local Authorities, based on structural parameters such as population density, commuting patterns and economic structure.

## Results

**Fig. 1:**
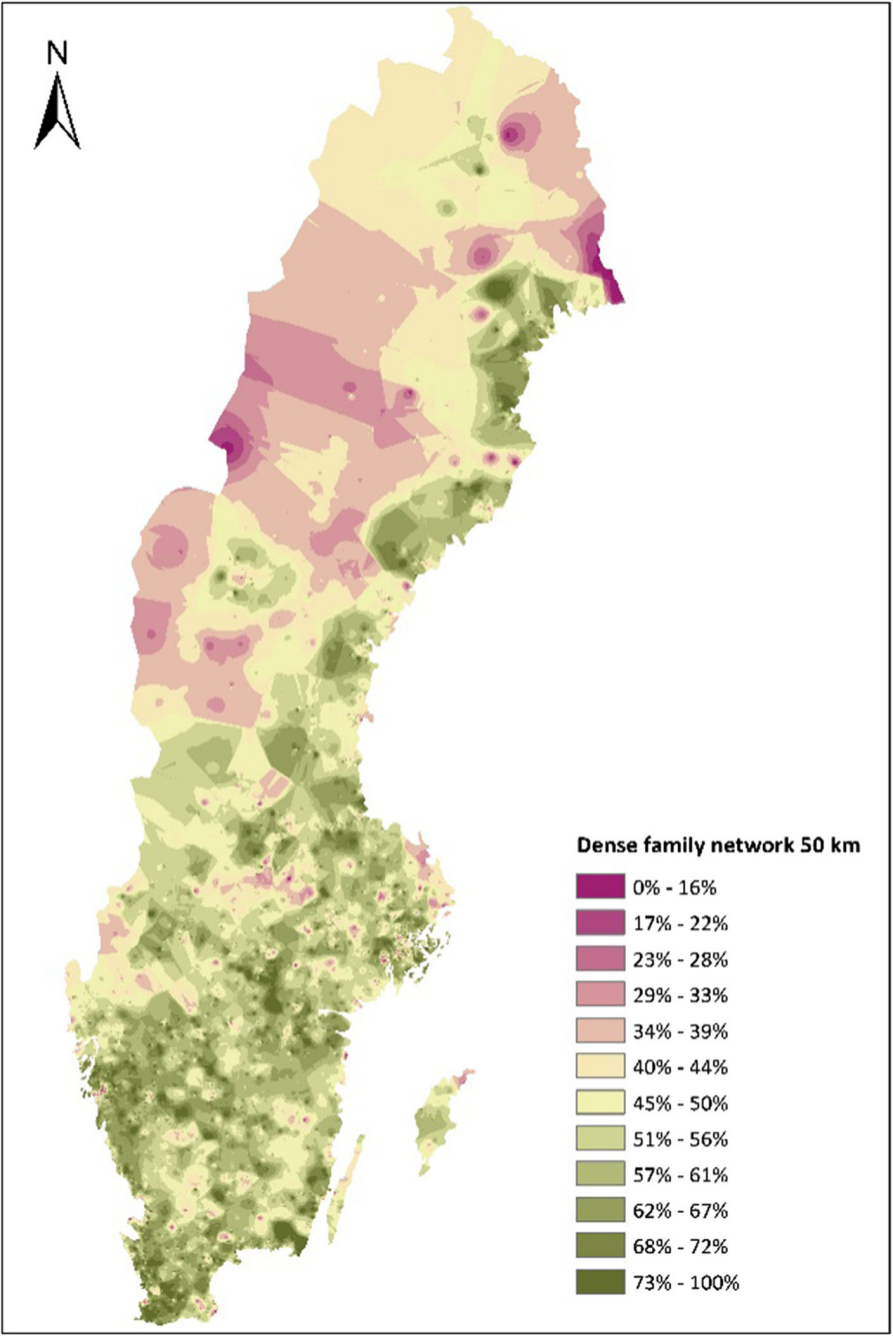
Density map of family networks for 60-year olds in Sweden

The density of family networks differs considerably across Sweden (Fig. 1). There is a clear tendency that the local networks are denser (including kin in both older/own generation and younger) in the metropolitan regions while local family networks are much sparser in the depopulated regions in the North West. There are also pockets of localities where a smaller proportion of the 60-year-olds have a dense family network. These are both regions that have suffered from depopulation such as the inner parts of southern Sweden or the manufacturing areas of mid-Sweden. Also, amenity-rich locations on the islands of Öland and Gotland and eastern part of Skåne in the south are areas where a significant proportion of the 60-year olds lack a local family network, supposedly because they have moved to the region, away from their kin.

**Fig. 2:**
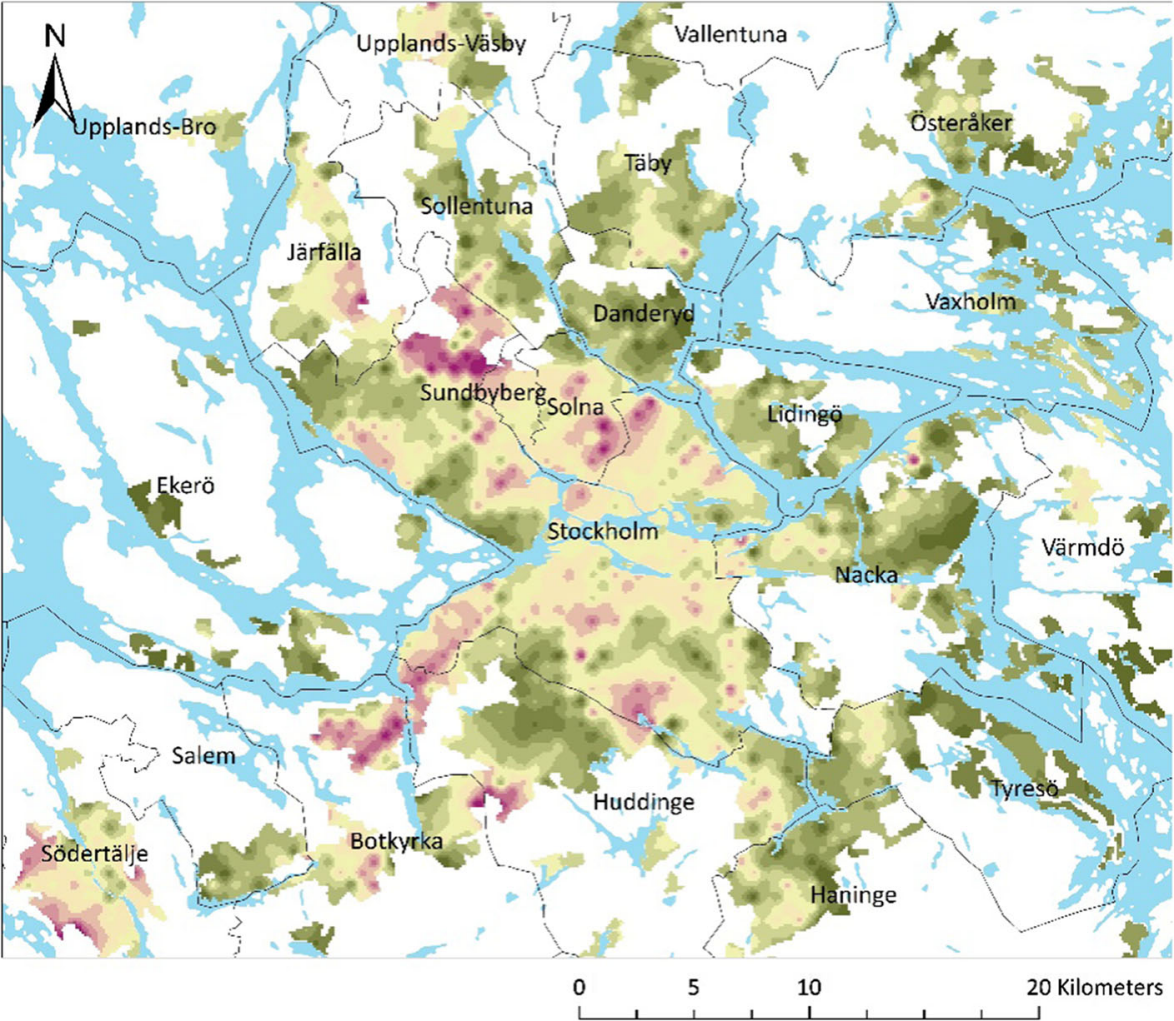
Density map of family networks for 60-year olds in Stockholm

Notably there are also significant variations on a much smaller scale, down to the neighbourhood level (Fig. 2). In the Stockholm area we see neighbourhoods where almost all 60-year olds have family members in younger and older generations within 50 km, while a neighbouring area is inhabited by 60 year olds where the majority does not have such dense networks. Some areas with less dense family networks include suburbs with a high share of immigrants. There is a strong correlation between share of immigrants in the neighbourhoods^1^ and share of persons with dense networks (r^2^ = 0.19). This could be explain the lack of family listed in the registers but also reflects a real lack of local family networks. The reason why fewer immigrants have dense family networks in both older and younger generations is mainly an effect of absent parents and siblings, while the difference when it comes to proximity to adult children is much smaller between Swedish born and immigrants (74 % compared to 71 %). In the wealthiest suburbs, like Täby, Danderyd and Lidingö, inhabitants tend to be rich, not only in terms of economic capital, but also in term of access to dense local family networks.

**Fig. 3:**
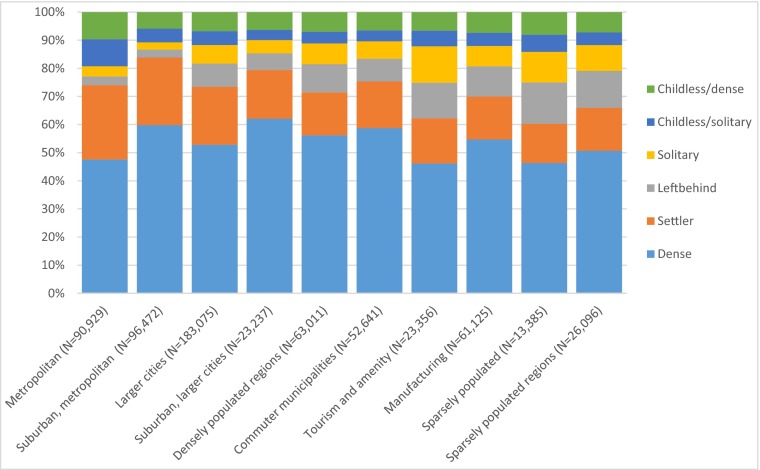
Distribution of family network category in different types of municipalities

Figure 3 illustrates that 60-year olds who are in different categories of local family networks differ considerably in different types of municipalities. Metropolitan areas have the highest share of childless adults in this age group: one out of five do not have adult children. This category is twice as common in metropolitan municipalities as compared to suburban municipalities. In the childless category, about half have no local family network, neither parents nor siblings. This makes this a potentially vulnerable group. Although metropolitan areas have the highest share of childless 60-year olds, those who do have adult children are very likely to live within the same region. The densest local family networks are found in the suburban municipalities of the metropolitan areas. The categories with absent adult children (solitary and left behinds) are uncommon among 60-year olds in these parts of the country and constitute only six percent when combined.

**Fig. 4:**
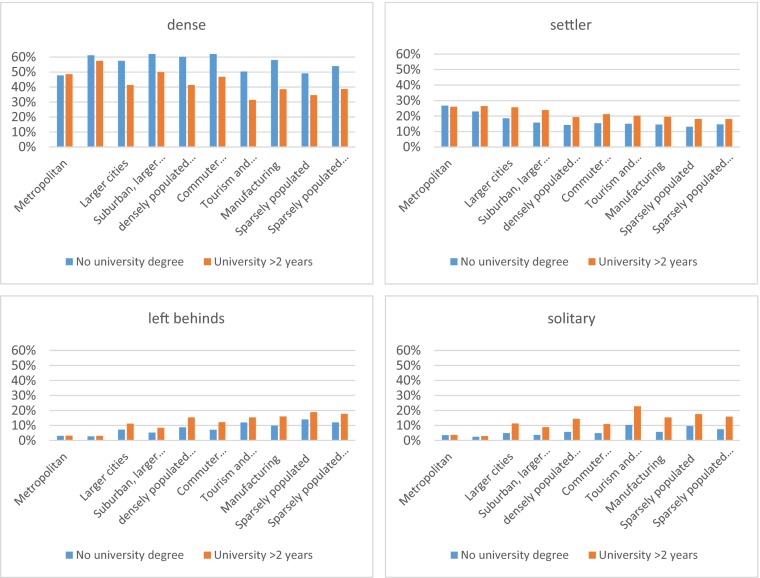
Type of local family network and education level

In the typical tourism and amenity municipalities and the sparsely populated municipalities, we find the lowest share of dense local family networks. Here we find the highest share of solitary 60-year olds who have no local family network, and also a relatively large share of left behinds. In these municipalities, one out of four 60-year olds belong to either of these categories (Fig. 4)

Recent migration among the 60-year-olds was defined as living in another functional region (FA-regions) compared to age 55. Since this is a sedentary age category, only 3 % of the individuals were recent migrants. It was, however, not surprising to find that the share of people who changed region is considerably higher in the solitary category, meaning those parents living far from both children and parents/siblings. In the solitary category, as many people as 14 % had moved over the past 5 years.

People with lower educational attainment are more likely to have a dense local family network in all municipality types except metropolitan areas where there is no significant difference when considering education levels. In all regions outside the metropolitan areas, those with higher education are more likely to have a less dense local family networks i.e., being left behinds, settlers or solitary. The differences between education levels are generally very small in all categories for metropolitan regions.

## Summary and Conclusions

Although this empirical study is merely a snapshot, the interpretation of the results implies a life course perspective which shows that events in life have long-term effects on conditions later in life. The landscape of local family networks is shaped by migration events throughout the life course, both their own migration and the migration undertaken by other family members. Therefore it is important not only to consider the individual life course but also to apply a family life course perspective.

The long-term spatial outcome of internal and international migration produces diverse preconditions for care and support from family members in older age in different regional contexts. Thereby, this has an impact on the living conditions in old age. The classification of the local family networks developed in this paper; including dense networks, settlers, left behinds and solitaires, is an attempt not only to describe the different groups but also to emphasise the underlying migration processes. In general, most 60-year-olds in Sweden have access to a local family network. The most common state is living in a dense family network with family members both in their own and/or parental generation and adult children within a day’s travel (50 km). There are, however, regional variations and these patterns are important in understanding future care burdens in different regions as well as the living conditions for both older and younger generations. Urban families in Sweden are more concentrated and have better geographic preconditions to encourage interaction. The access to local family networks not only varies on a broad rural–urban scale but also locally, such between neighbourhoods within metropolitan areas. In some urban localities (especially in some suburban parts) it seems that almost all parents in this age group have a dense local family network while nearby neighbourhoods are characterised by large groups of 60-year olds lacking local family networks.

Mobility generates these patterns and since we know that people with higher educational achievements are more mobile and that their children are more likely to enrol in higher education and thereby move away, we expect the family networks of people with higher education to be more dispersed. One conclusion from this study is that this is not true in all geographical contexts. Among 60-year olds in metropolitan areas, the highly educated are just as likely as people with lower educational achievements to have a dense family network.

Families in metropolitan areas are the most concentrated geographically, and parents who grow old in metropolitan areas seems to have better preconditions for relying on informal care and assistance in later life. However, in terms of access to family networks, there is a vulnerable group that is larger in the metropolitan areas compared to other regions and that is the childless. This group constitutes 20 % in metropolitan areas, of which half does not have a local family network at all. Another vulnerable group is immigrants who often lack horizontal ties to siblings and vertical links to parents in Sweden. Further research is needed to scrutinise these two groups weak position when it comes to access to family networks.

As expected, these networks have different characteristics in rural and urban areas. The left behind parent, embedded in a local network in their own and older generation, is a small category in urban areas but quite common in some rural municipalities. A high concentration of solitary elderly people with a geographically dispersed family over all generations is concentrated in some typical amenity and tourism municipalities. This illustrates how mobility processes in a life course perspective shape the geography of families, in this case migration later in life. In this category we find an overrepresentation of recent internal migrants.

This study can only identify the preconditions for interaction between family members in terms of distance. The quality of the relationship and the frequency of contact cannot be ascertained through register data. We cannot tell whether family bonds are stronger in rural areas compared to urban, but what we can tell that elderly people in rural areas are more likely to find themselves in a situation where their family members live at a distance that prohibits day to day physical interaction and face-to-face contact. Since geographic distance hinders daily interaction, in these cases there is no latent local family network that can step in if assistance is required.

It is important to acknowledge geographical aspects for the demographic process of ageing. As a population ages, it puts strain on the welfare state, therefore, families might become the most important providers of care and support for the elderly. It is crucial to bear in mind that access to this care is restrained by geographical distance, and this pertains more so for certain groups and in some regions rather than others. In conclusion, internal and international migration processes have a long-term impact on the availability of care and support in an ageing population.

## Footnotes

1 Neighbourhood is here defined as 1 km × 1 km grid.

## References

[CR1] Albertini M, Kohli M, Vogel C (2007). Intergenerational transfers of time and money in European families: common patterns different regimes?. Journal of European Social Policy.

[CR2] Bell D, Rutherford A (2013). Individual and geographic factors in the formation of care networks in the UK. Population Space and Place.

[CR3] Bengtson VL (2001). Beyond the nuclear family: the increasing importance of multigenerational bonds. Journal of Marriage and Family.

[CR4] Blaauboer M, Stjernström O, Strömgren M (2013). Life course preferences, sibling ties, and the geographical dispersion of sibling networks. Population Space and Place.

[CR5] Chan, T.W., & Ermisch, J. (2011). Intergenerational exchange of instrumental support: dynamic evidence from the british household panel survey. In Sociology Working Papers University of Oxford.

[CR6] de Jong Gierveld J, Dykstra PA (2013). Virtue is its own reward? Support-giving in the family and loneliness in middle and old age. Ageing and Society.

[CR7] Duncan S, Smith D (2002). Geographies of family formations: spatial differences and gender cultures in Britain. Transactions of the Institute of British Geographers.

[CR8] Elder GH (1994). Time, human agency, and social change: perspectives on the life course. Social Psychology Quarterly.

[CR9] Fischer P, Malmberg G (2001). Settled people don’t move: on life course and (Im-) mobility in Sweden. International Journal of Population Geography.

[CR10] Fors S, Lennartsson C (2008). Social mobility, geographical proximity and intergenerational family contact in Sweden. Ageing and Society.

[CR11] Friedrich K, Warnes T (2000). Understanding contrasts in later life migration patterns: Germany, Britain and the United States. Erdkunde.

[CR12] Halleröd, B. (2006). Resursomfördelning mellan generationer: utbyte av gåvor och tjänster mellan föräldrar och barn. In J. Vogel and L. Häll (Eds.), Äldres levnadsförhållanden. Arbete, ekonomi, hälsa och sociala nätverk 1980–2003 (Vol. 112, pp. 257–270). Örebro: Statistiska centralbyrån.

[CR13] Hank K (2007). Proximity and contacts between older parents and their children: a European comparison. Journal of Marriage and Family.

[CR14] He, C., & Ye, J. (2013). Lonely sunsets: impacts of rural–urban migration on the left‐behind elderly in rural China. Population, Space and Place, published online: 15 OCT 2013.

[CR15] Hjälm, A. (2011). A family landscape. On the geographical distances between elderly parents and adult children in Sweden. Umeå University.

[CR16] Hoff A (2007). Patterns of intergenerational support in grandparent-grandchild and parent–child relationships in Germany. Ageing and Society.

[CR17] Keating N, Swindle J, Fletcher S (2011). Aging in rural Canada: a retrospective and review. Canadian Journal on Aging/La Revue canadienne du vieillissement.

[CR18] Knodel J, Saengtienchai C (2007). Rural parents with urban children: social and economic implications of migration for the rural elderly in Thailand. Population Space and Place.

[CR19] Lundholm E (2007). Are movers still the same? Characteristics of interregional migrants in Sweden 1970–2001. Tijdschrift Voor Economische en Sociale Geografie.

[CR20] Lundholm, E. (2012a). Return to where? The geography of elderly return migration in Sweden. European Urban and Regional Studies, Published online before print.

[CR21] Lundholm E (2012). Returning home? Migration to birthplace among migrants after age 55. Population Space and Place.

[CR22] Lundholm E, Garvill J, Malmberg G, Westin K (2004). Forced or free movers? The motives, voluntariness and selectivity of interregional migration in the Nordic countries. Population Space and Place.

[CR23] Malmberg G, Pettersson A (2008). Distance to elderly parents: analyses of Swedish register data. Demographic Research.

[CR24] Michielin F, Mulder C (2007). Geographical distances between adult children and their parents in the Netherlands. Demographic Research.

[CR25] Mulder CH, van der Meer MJ (2009). Geographical distances and support from family members. Population Space and Place.

[CR26] Pettersson A, Malmberg G (2009). Adult children and elderly parents as mobility attractions in Sweden. Population Space and Place.

[CR27] Rainer H, Siedler T (2012). Family location and caregiving patterns from an international perspective. Population and Development Review.

[CR28] Scharf T (2001). Ageing and intergenerational relationships in rural Germany. Ageing and Society.

[CR29] Smith DP (2011). Geographies of long-distance family migration: moving to a ‘spatial turn’. Progress in Human Geography.

[CR30] Statistics Sweden (2010). Multi-generation register 2009. A description of contents and quality.

[CR31] Stockdale A (2006). The role of a ‘retirement transition’ in the repopulation of rural areas. Population Space and Place.

[CR32] Sundström G, Malmberg B, Johansson L (2006). Balancing family and state care: neither, either or both? The case of Sweden. Ageing and Society.

[CR33] Szebehely, M. (2006). Informella hjälpgivare. In J. Vogel and L. Häll (Eds.), Äldres levnadsförhållanden. Arbete, ekonomi, hälsa och sociala nätverk 1980–2003 (Vol. 112, pp. 435–461). Örebro: Statistiska centralbyrån.

[CR34] Szebehely M, Trydegård G-B (2012). Home care for older people in Sweden: a universal model in transition. Health and Social Care in the Community.

[CR35] van der Pers M, Mulder CH (2013). The regional dimension of intergenerational proximity in the Netherlands. Population Space and Place.

[CR36] van Gaalen, R. I., Dykstra, P. A., Flap, H. (2008). Intergenerational contact beyond the dyad: the role of the sibling network. European Journal of Ageing, 5. 10.1007/s10433-008-0076-6 PMC5546385 28798559

[CR37] Voorpostel M, van der Lippe T, Dykstra P, Flap H (2007). Similar or different? The importance of similarities and differences for support between siblings. Journal of Family Issues.

[CR38] Voorpostel M, Lippe TVD, Flap H (2012). For better or worse: negative life events and sibling relationships. International Sociology.

[CR39] Vullnetari J, King R (2008). ‘Does your granny eat grass?’ On mass migration, care drain and the fate of older people in rural Albania. Global Networks.

[CR40] Walford NS, Kurek S (2008). A comparative analysis of population ageing in urban and rural areas of England and Wales, and Poland over the last three census intervals. Population Space and Place.

[CR41] Wanless D, Mitchell BA, Wister AV (2010). Social determinants of health for older women in Canada: does rural–urban residency matter?. Canadian Journal on Aging.

[CR42] Wenger GC (2001). Myths and realities of ageing in rural Britain. Ageing and Society.

